# Absolute configuration of isoeichlerialactone

**DOI:** 10.1107/S1600536810009499

**Published:** 2010-03-20

**Authors:** Hoong-Kun Fun, Nantiya Joycharat, Supayang Piyawan Voravuthikunchai, Suchada Chantrapromma

**Affiliations:** a X-ray Crystallography Unit, School of Physics, Universiti Sains Malaysia, 11800 USM, Penang, Malaysia; b Natural Products Research Center, Faculty of Science, Prince of Songkla University, Hat-Yai, Songkhla 90112, Thailand; c Crystal Materials Research Unit, Department of Chemistry, Faculty of Science, Prince of Songkla University, Hat-Yai, Songkhla 90112, Thailand

## Abstract

The title seco-dammarane triterpenoid, C_27_H_42_O_4_ (systematic name: 3-{(3*S*,3a*R*,5a*R*,6*S*,7*S*,9a*R*,9b*R*)-6,9a,9b-trimethyl-3-[(*R*)-2-methyl-5-oxotetra­hydro­furan-2-yl]-7-(prop-1-en-2-yl)dodeca­hydro-1*H*-cyclo­penta­[*a*]naphthalen-6-yl}propanoic acid), has been isolated for the first time from the seeds of *Aglaia forbesii*. The mol­ecule has three fused rings and all rings are in *trans*-fused. The two cyclo­hexane rings are in standard chair conformations and the cyclo­pentane ring adopts an envelope conformation. Its absolute configuration was determined by the refinement of the Flack parameter to 0.26 (17). In the crystal, mol­ecules are linked into chains along [010] by O—H⋯O hydrogen bonds.

## Related literature

For details of ring conformations, see: Cremer & Pople (1975 ▶). For bond-length data, see: Allen *et al.* (1987 ▶). For background to triterpenes and their biological activity, see: Engelmeier *et al.* (2000 ▶); Greger *et al.* (2001 ▶); Joycharat *et al.* (2008 ▶, 2010 ▶); Kim *et al.* (2006 ▶); Proksch *et al.* (2005 ▶). For a related structure, see: Singh & Aalbersberg (1992 ▶). For the stability of the temperature controller used in the data collection, see: Cosier & Glazer, (1986 ▶).
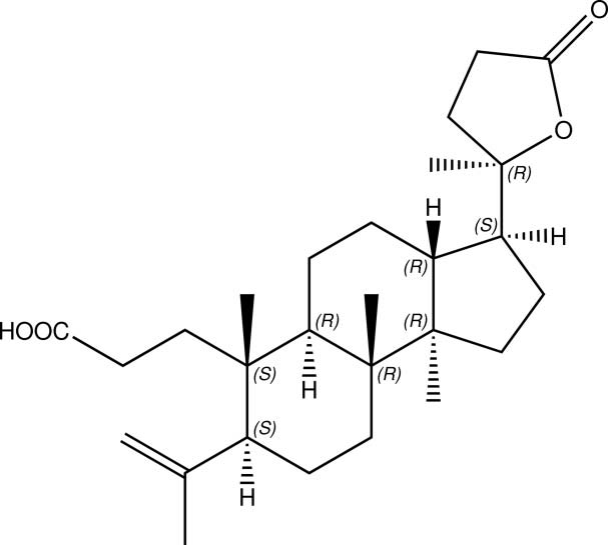


## Experimental

### 

#### Crystal data

C_27_H_42_O_4_
*M* *_r_* = 430.61
Monoclinic, 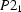

*a* = 11.8690 (4) Å
*b* = 7.0388 (3) Å
*c* = 14.1173 (5) Å
β = 94.962 (2)°
*V* = 1174.99 (8) Å^3^
*Z* = 2
Cu *K*α radiation
μ = 0.63 mm^−1^
*T* = 100 K
0.56 × 0.14 × 0.06 mm

#### Data collection

Bruker APEX DUO CCD area-detector diffractometer
Absorption correction: multi-scan (*SADABS*; Bruker, 2009 ▶) *T*_min_ = 0.720, *T*_max_ = 0.962
29402 measured reflections
3253 independent reflections
3209 reflections with *I* > 2σ(*I*)
*R*_int_ = 0.045

#### Refinement

*R*[*F* ^2^ > 2σ(*F* ^2^)] = 0.029
*wR*(*F* ^2^) = 0.076
*S* = 1.05
3253 reflections
286 parameters
1 restraint
H-atom parameters constrained
Δρ_max_ = 0.20 e Å^−3^
Δρ_min_ = −0.19 e Å^−3^
Absolute structure: Flack (1983 ▶), 1258 Friedel pairs
Flack parameter: 0.26 (17)

### 

Data collection: *APEX2* (Bruker, 2009 ▶); cell refinement: *SAINT* (Bruker, 2009 ▶); data reduction: *SAINT*; program(s) used to solve structure: *SHELXTL* (Sheldrick, 2008 ▶); program(s) used to refine structure: *SHELXTL*; molecular graphics: *SHELXTL*; software used to prepare material for publication: *SHELXTL* and *PLATON* (Spek, 2009 ▶).

## Supplementary Material

Crystal structure: contains datablocks global, I. DOI: 10.1107/S1600536810009499/rz2425sup1.cif

Structure factors: contains datablocks I. DOI: 10.1107/S1600536810009499/rz2425Isup2.hkl

Additional supplementary materials:  crystallographic information; 3D view; checkCIF report

## Figures and Tables

**Table 1:** Hydrogen-bond geometry (Å, °)

*D*—H⋯*A*	*D*—H	H⋯*A*	*D*⋯*A*	*D*—H⋯*A*
O2—H1*O*2⋯O1^i^	0.82	1.88	2.6541 (18)	156

Symmetry code: (i) 
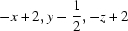
.

**Fig. 1.**
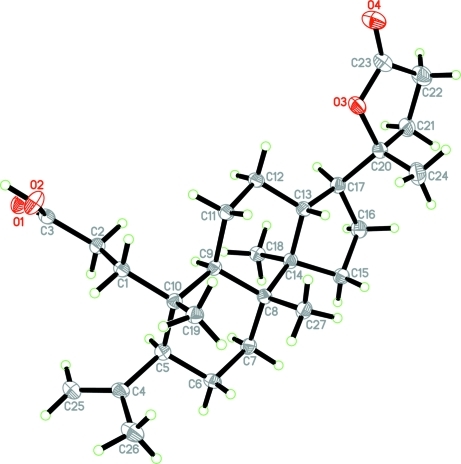
The structure of the title compound, showing 50% probability displacement ellipsoids and the atom-numbering scheme.

**Fig. 2.**
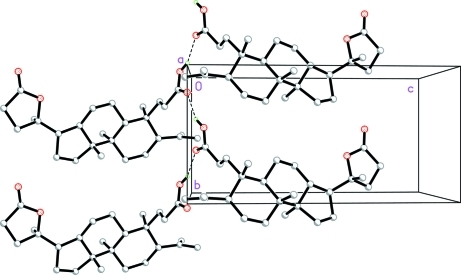
The crystal packing of the title compound viewed along the a axis, showing a chain of molecules along the [010] direction formed by intermolecular O—H···O hydrogen bonds (dashed lines). H atoms not involved in hydrogen bonding have been omitted for clarity.

## supplementary crystallographic information

### Comment

The genus *Aglaia* is a rich source of a number of interesting constituents, such as flavaglines, bisamides, triterpenoids, and limonoids which have insecticidal, antifungal, anti-inflammatory and cytotoxic activities (Engelmeier *et al.*, 2000; Greger *et al*., 2001; Kim *et al.*, 2006; Proksch *et al.*, 2005). *Aglaia forbesii* is a large tree mainly distributed in southern Thailand. Previous phytochemical studies on the leaves of *Aglaia forbesii* showed that some of the isolated compounds from this plant showed antituberculosis and antiviral activities (Joycharat *et al.*, 2008). The title seco-dammarane triterpenoid was isolated for the first time from the seeds of *Aglaia forbessi* which was collected from Nakhon Si Thammarat province in the southern part of Thailand. Its absolute configuration was determined by making use of the anomalous scattering of Cu Kα*X*-radiation with the the Flack parameter refined to 0.26 (17). We report herein the crystal structure of the title compound.

Fig. 1 shows that the molecule of the title compound has three fused rings and all rings are in *trans*-fused. The two cyclohexane rings are in standard chair conformations. The cyclopentane (C13–C17) ring adopts an envelope conformation with the puckered C14 atom having the maximum deviation of 0.2865 (19) Å, *Q* =  0.45556 (19) Å and θ = 222.1 (2)° whereas the furan ring (C20–C23/O3) is twisted with the C20 and C21 atoms having deviations of -0.169 (2) and 0.185 (2) Å, respectively from the C22/C23/O3 plane, with *Q* =  0.297 (2) Å and θ = 61.9 (4)° (Cremer & Pople, 1975). Atoms C2, C3, O1 and O2 of the propanoic acid lie on the same plane with r.m.s. deviation of 0.0050 (2) Å. The orientation of the propanoic acid [C1–C3/O1–O2] group is described by the torsion angles C10–C1–C2–C3 = 179.42 (14)°, C1–C2–C3–O1 = -167.70 (14)° and C1–C2–C3–O2 = 14.2 (2)°. The bond angles around C4 and C25 atoms are indicative of *sp*^2^ hybridization for these atoms and the bond length of 1.374 (3) Å confirms the C4═C25 bond. All bond distances are within normal ranges (Allen *et al.*, 1987). The configurations at atoms C5, C8, C9, C10, C13, C14, C17 and C20 are in *S*, *R*, *R*, *S*, *R*, *R*, *S* and *R*, respectively. In the title compound (isoeichlerialactone), the configuration at atom C20 (Fig. 1) or position 2 of the 2-methyl-5-oxotetrahydrofuran unit [C20–C24/O3–O4] was established as *R*-methyl configuration whereas in the eichlerialactone (Singh & Aalbersberg, 1992), this position is in *S*-configuration and the remaining positions are in the same configurations.

In the crystal packing (Fig. 2), the molecules are arranged into one dimensional chains along the *b* axis by intermolecular O—H···O hydrogen bonds involving O atoms of propanoic acid groups (Table 1).

### Experimental

The seeds of *Aglaia forbesii* (48 g) were air-dried and ground, and exhaustively extracted with EtOH (3 × 500 *mL*) at room temperature. The combined extracts were concentrated under reduced pressure to afford a brown extract (5.7 g) which was resuspended in a mixture of MeOH and water and then extracted with n-hexane, CH_2_Cl_2_, and BuOH, successively. The CH_2_Cl_2_ fraction (1.87 g) was applied to column chromatography over silica gel (Merck, 0.063-0.200 mm) using gradient elution from 2% to 100% acetone in CH_2_Cl_2_, and finally washed down with MeOH. The fraction eluted with 12% acetone in CH_2_Cl_2_ was further purified on columns of silica gel (CH_2_Cl_2_-acetone, 92:8 *v*/*v*) and Sephadex LH20 (CH_2_Cl_2_-MeOH, 1:1 *v*/*v*) to yield the title compound (10.7 mg). Colourless needle-shaped single crystals of the title compound suitable for *X*-ray structure determination were recrystallized from EtOH after several days. ^1^H NMR and ^13^C NMR spectral data (Joycharat *et al.*, 2010) were consistent with the *X*-ray structure.

### Refinement

After confirming their positions from the difference map, all H atoms were placed in calculated positions with d(O—H) = 0.82 Å and d(C—H) = 0.93 Å for aromatic, 0.97 for CH_2_ and 0.96 Å for CH_3_ atoms. The *U*_iso_ values were constrained to be 1.5*U*_eq_ of the carrier atom for hydroxy and methyl H atoms and 1.2*U*_eq_ for the remaining H atoms. A rotating group model was used for the methyl groups. The highest residual electron density peak is located at 0.67 Å from H26B and the deepest hole is located at 1.15 Å from C12. 1258 Friedel pairs were used to determine the absolute configuration.

### Crystal data

**Table:**

C_27_H_42_O_4_	*F*(000) = 472
*M**_r_* = 430.61	*D*_x_ = 1.217 Mg m^−^^3^
Monoclinic, *P*2_1_	Cu *K*α radiation, λ = 1.54178 Å
Hall symbol: P 2yb	Cell parameters from 3253 reflections
*a* = 11.8690 (4) Å	θ = 3.1–63.5°
*b* = 7.0388 (3) Å	µ = 0.63 mm^−^^1^
*c* = 14.1173 (5) Å	*T* = 100 K
β = 94.962 (2)°	Needle, colourless
*V* = 1174.99 (8) Å^3^	0.56 × 0.14 × 0.06 mm
*Z* = 2	

### Data collection

**Table:**

Bruker APEX DUO CCD area-detector diffractometer	3253 independent reflections
Radiation source: sealed tube	3209 reflections with *I* > 2σ(*I*)
graphite	*R*_int_ = 0.045
φ and ω scans	θ_max_ = 63.5°, θ_min_ = 3.1°
Absorption correction: multi-scan (*SADABS*; Bruker, 2009)	*h* = −13→13
*T*_min_ = 0.720, *T*_max_ = 0.962	*k* = −6→7
29402 measured reflections	*l* = −16→16

### Refinement

**Table:**

Refinement on *F*^2^	Hydrogen site location: inferred from neighbouring sites
Least-squares matrix: full	H-atom parameters constrained
*R*[*F*^2^ > 2σ(*F*^2^)] = 0.029	*w* = 1/[σ^2^(*F*_o_^2^) + (0.0429*P*)^2^ + 0.3283*P*] where *P* = (*F*_o_^2^ + 2*F*_c_^2^)/3
*wR*(*F*^2^) = 0.076	(Δ/σ)_max_ = 0.001
*S* = 1.05	Δρ_max_ = 0.20 e Å^−^^3^
3253 reflections	Δρ_min_ = −0.19 e Å^−^^3^
286 parameters	Extinction correction: *SHELXTL* (Sheldrick, 2008), Fc^*^=kFc[1+0.001xFc^2^λ^3^/sin(2θ)]^-1/4^
1 restraint	Extinction coefficient: 0.0060 (7)
Primary atom site location: structure-invariant direct methods	Absolute structure: Flack (1983), 1258 Friedel pairs
Secondary atom site location: difference Fourier map	Flack parameter: 0.26 (17)

### Special details

**Table:**

Experimental. The crystal was placed in the cold stream of an Oxford Cryosystems Cobra open-flow nitrogen cryostat (Cosier & Glazer, 1986) operating at 100.0 (1) K.
Geometry. All esds (except the esd in the dihedral angle between two l.s. planes) are estimated using the full covariance matrix. The cell esds are taken into account individually in the estimation of esds in distances, angles and torsion angles; correlations between esds in cell parameters are only used when they are defined by crystal symmetry. An approximate (isotropic) treatment of cell esds is used for estimating esds involving l.s. planes.
Refinement. Refinement of F^2^ against ALL reflections. The weighted R-factor wR and goodness of fit S are based on F^2^, conventional R-factors R are based on F, with F set to zero for negative F^2^. The threshold expression of F^2^ > 2sigma(F^2^) is used only for calculating R-factors(gt) etc. and is not relevant to the choice of reflections for refinement. R-factors based on F^2^ are statistically about twice as large as those based on F, and R- factors based on ALL data will be even larger.

### Fractional atomic coordinates and isotropic or equivalent isotropic displacement parameters (Å^2^)

**Table:**

	*x*	*y*	*z*	*U*_iso_*/*U*_eq_	
O1	1.02039 (9)	0.12899 (19)	0.98069 (8)	0.0230 (3)	
O2	0.87449 (10)	−0.06457 (19)	0.94649 (9)	0.0269 (3)	
H1O2	0.9144	−0.1370	0.9802	0.040*	
O3	0.85383 (10)	0.17561 (19)	0.33396 (8)	0.0233 (3)	
O4	0.92934 (11)	−0.0534 (2)	0.24954 (9)	0.0311 (3)	
C1	0.73873 (13)	0.1943 (3)	0.85206 (11)	0.0165 (4)	
H1A	0.7030	0.1786	0.9108	0.020*	
H1B	0.7359	0.0721	0.8202	0.020*	
C2	0.86329 (13)	0.2436 (3)	0.87797 (11)	0.0182 (4)	
H2A	0.9005	0.2569	0.8198	0.022*	
H2B	0.8674	0.3656	0.9101	0.022*	
C3	0.92647 (13)	0.0998 (3)	0.94034 (11)	0.0181 (4)	
C4	0.60276 (14)	0.5092 (3)	0.93919 (11)	0.0220 (4)	
C5	0.65278 (13)	0.5261 (3)	0.84351 (11)	0.0180 (4)	
H5A	0.7296	0.5754	0.8576	0.022*	
C6	0.58968 (13)	0.6758 (3)	0.78086 (11)	0.0183 (4)	
H6A	0.5825	0.7912	0.8174	0.022*	
H6B	0.5142	0.6301	0.7612	0.022*	
C7	0.65149 (14)	0.7207 (3)	0.69250 (11)	0.0190 (4)	
H7A	0.7241	0.7776	0.7125	0.023*	
H7B	0.6078	0.8131	0.6539	0.023*	
C8	0.67072 (12)	0.5444 (3)	0.63145 (11)	0.0162 (4)	
C9	0.72743 (13)	0.3854 (3)	0.69732 (11)	0.0150 (4)	
H9A	0.8000	0.4401	0.7222	0.018*	
C10	0.66594 (13)	0.3357 (3)	0.78849 (11)	0.0163 (4)	
C11	0.75991 (13)	0.2106 (3)	0.64025 (11)	0.0178 (4)	
H11A	0.6915	0.1505	0.6120	0.021*	
H11B	0.7987	0.1198	0.6833	0.021*	
C12	0.83640 (13)	0.2598 (3)	0.56117 (11)	0.0182 (4)	
H12A	0.9090	0.3055	0.5890	0.022*	
H12B	0.8492	0.1474	0.5238	0.022*	
C13	0.77909 (13)	0.4124 (3)	0.49785 (11)	0.0164 (4)	
H13A	0.7055	0.3611	0.4733	0.020*	
C14	0.75490 (12)	0.5929 (3)	0.55434 (11)	0.0167 (4)	
C15	0.71234 (13)	0.7261 (3)	0.47270 (11)	0.0195 (4)	
H15A	0.7126	0.8574	0.4936	0.023*	
H15B	0.6365	0.6920	0.4472	0.023*	
C16	0.79861 (14)	0.6939 (3)	0.39867 (12)	0.0230 (4)	
H16A	0.8625	0.7794	0.4101	0.028*	
H16B	0.7638	0.7156	0.3349	0.028*	
C17	0.83734 (13)	0.4841 (3)	0.41098 (11)	0.0185 (4)	
H17A	0.9192	0.4831	0.4275	0.022*	
C18	0.86642 (13)	0.6806 (3)	0.59992 (11)	0.0185 (4)	
H18A	0.9253	0.6623	0.5583	0.028*	
H18B	0.8872	0.6198	0.6598	0.028*	
H18C	0.8558	0.8141	0.6099	0.028*	
C19	0.55240 (13)	0.2318 (3)	0.76682 (11)	0.0184 (4)	
H19A	0.5312	0.1710	0.8235	0.028*	
H19B	0.5600	0.1379	0.7185	0.028*	
H19C	0.4952	0.3218	0.7450	0.028*	
C20	0.81270 (14)	0.3722 (3)	0.31846 (11)	0.0198 (4)	
C21	0.88153 (15)	0.4451 (3)	0.23872 (12)	0.0256 (4)	
H21A	0.8388	0.5371	0.1991	0.031*	
H21B	0.9519	0.5027	0.2644	0.031*	
C22	0.90316 (16)	0.2656 (3)	0.18336 (13)	0.0312 (5)	
H22A	0.8455	0.2481	0.1311	0.037*	
H22B	0.9767	0.2701	0.1583	0.037*	
C23	0.89845 (14)	0.1082 (3)	0.25590 (12)	0.0246 (4)	
C24	0.68744 (15)	0.3543 (3)	0.28607 (12)	0.0306 (5)	
H24A	0.6503	0.2829	0.3322	0.046*	
H24B	0.6786	0.2902	0.2259	0.046*	
H24C	0.6544	0.4787	0.2797	0.046*	
C25	0.67355 (16)	0.5249 (3)	1.02112 (12)	0.0328 (5)	
H25A	0.6438	0.5250	1.0799	0.039*	
H25B	0.7512	0.5354	1.0178	0.039*	
C26	0.48110 (15)	0.4927 (3)	0.94372 (13)	0.0288 (5)	
H26A	0.4654	0.4626	1.0076	0.043*	
H26B	0.4522	0.3938	0.9016	0.043*	
H26C	0.4455	0.6110	0.9250	0.043*	
C27	0.55534 (13)	0.4831 (3)	0.58199 (11)	0.0198 (4)	
H27A	0.4990	0.4881	0.6268	0.030*	
H27B	0.5608	0.3557	0.5585	0.030*	
H27C	0.5345	0.5673	0.5299	0.030*	

### Atomic displacement parameters (Å^2^)

**Table:**

	*U*^11^	*U*^22^	*U*^33^	*U*^12^	*U*^13^	*U*^23^
O1	0.0221 (6)	0.0222 (8)	0.0237 (6)	0.0001 (5)	−0.0039 (5)	−0.0002 (5)
O2	0.0239 (6)	0.0207 (8)	0.0348 (7)	−0.0002 (6)	−0.0052 (5)	0.0082 (6)
O3	0.0287 (6)	0.0248 (9)	0.0170 (6)	−0.0003 (5)	0.0044 (5)	−0.0021 (5)
O4	0.0385 (7)	0.0257 (10)	0.0308 (7)	−0.0002 (6)	0.0136 (5)	−0.0034 (6)
C1	0.0192 (8)	0.0153 (10)	0.0153 (7)	−0.0003 (7)	0.0036 (6)	−0.0009 (7)
C2	0.0209 (8)	0.0166 (11)	0.0172 (8)	0.0013 (7)	0.0019 (6)	0.0010 (7)
C3	0.0206 (8)	0.0183 (11)	0.0159 (8)	0.0003 (7)	0.0044 (6)	−0.0020 (7)
C4	0.0322 (9)	0.0147 (11)	0.0201 (8)	0.0067 (8)	0.0082 (7)	0.0009 (8)
C5	0.0178 (7)	0.0186 (11)	0.0176 (8)	−0.0009 (7)	0.0017 (6)	−0.0003 (7)
C6	0.0213 (8)	0.0156 (11)	0.0187 (8)	0.0005 (7)	0.0054 (6)	−0.0025 (7)
C7	0.0215 (8)	0.0166 (11)	0.0190 (8)	0.0019 (7)	0.0028 (6)	0.0036 (7)
C8	0.0172 (8)	0.0165 (11)	0.0150 (8)	0.0007 (7)	0.0024 (6)	0.0010 (7)
C9	0.0151 (7)	0.0138 (11)	0.0159 (7)	0.0004 (7)	0.0004 (6)	0.0006 (7)
C10	0.0161 (8)	0.0166 (11)	0.0161 (7)	0.0002 (7)	0.0015 (6)	0.0010 (7)
C11	0.0217 (8)	0.0154 (11)	0.0167 (8)	0.0007 (7)	0.0030 (6)	0.0007 (7)
C12	0.0210 (8)	0.0172 (11)	0.0169 (8)	0.0012 (7)	0.0040 (6)	−0.0022 (7)
C13	0.0165 (8)	0.0171 (11)	0.0157 (7)	−0.0001 (7)	0.0025 (6)	0.0005 (7)
C14	0.0164 (7)	0.0183 (11)	0.0156 (8)	−0.0003 (7)	0.0020 (6)	0.0016 (7)
C15	0.0226 (8)	0.0164 (11)	0.0198 (8)	0.0009 (7)	0.0028 (6)	0.0020 (7)
C16	0.0274 (9)	0.0225 (12)	0.0196 (8)	0.0005 (8)	0.0054 (7)	0.0049 (8)
C17	0.0167 (8)	0.0223 (12)	0.0165 (8)	−0.0003 (7)	0.0026 (6)	0.0011 (7)
C18	0.0206 (8)	0.0156 (11)	0.0197 (8)	−0.0020 (7)	0.0035 (6)	0.0001 (7)
C19	0.0187 (8)	0.0181 (11)	0.0187 (8)	−0.0004 (7)	0.0027 (6)	0.0003 (7)
C20	0.0225 (8)	0.0200 (12)	0.0168 (8)	0.0019 (7)	0.0018 (6)	0.0010 (7)
C21	0.0277 (9)	0.0305 (13)	0.0190 (8)	0.0014 (8)	0.0044 (7)	0.0027 (8)
C22	0.0354 (10)	0.0360 (14)	0.0237 (9)	−0.0007 (9)	0.0104 (7)	0.0009 (9)
C23	0.0261 (9)	0.0264 (14)	0.0219 (9)	−0.0034 (9)	0.0057 (7)	−0.0053 (8)
C24	0.0253 (9)	0.0462 (15)	0.0200 (8)	−0.0015 (9)	−0.0007 (7)	−0.0009 (9)
C25	0.0342 (10)	0.0465 (15)	0.0182 (8)	0.0131 (9)	0.0052 (7)	−0.0009 (9)
C26	0.0360 (10)	0.0268 (13)	0.0253 (9)	−0.0012 (9)	0.0122 (7)	−0.0018 (8)
C27	0.0169 (8)	0.0236 (12)	0.0190 (8)	0.0008 (7)	0.0015 (6)	0.0038 (7)

### Geometric parameters (Å, °)

**Table:**

O1—C3	1.2246 (19)	C13—C14	1.540 (3)
O2—C3	1.318 (2)	C13—C17	1.544 (2)
O2—H1O2	0.8200	C13—H13A	0.9800
O3—C23	1.349 (2)	C14—C15	1.537 (2)
O3—C20	1.478 (2)	C14—C18	1.549 (2)
O4—C23	1.201 (3)	C15—C16	1.542 (2)
C1—C2	1.532 (2)	C15—H15A	0.9700
C1—C10	1.552 (2)	C15—H15B	0.9700
C1—H1A	0.9700	C16—C17	1.552 (3)
C1—H1B	0.9700	C16—H16A	0.9700
C2—C3	1.500 (2)	C16—H16B	0.9700
C2—H2A	0.9700	C17—C20	1.532 (2)
C2—H2B	0.9700	C17—H17A	0.9800
C4—C25	1.374 (3)	C18—H18A	0.9600
C4—C26	1.456 (2)	C18—H18B	0.9600
C4—C5	1.526 (2)	C18—H18C	0.9600
C5—C6	1.530 (2)	C19—H19A	0.9600
C5—C10	1.564 (3)	C19—H19B	0.9600
C5—H5A	0.9800	C19—H19C	0.9600
C6—C7	1.534 (2)	C20—C24	1.522 (2)
C6—H6A	0.9700	C20—C21	1.535 (2)
C6—H6B	0.9700	C21—C22	1.520 (3)
C7—C8	1.539 (3)	C21—H21A	0.9700
C7—H7A	0.9700	C21—H21B	0.9700
C7—H7B	0.9700	C22—C23	1.513 (3)
C8—C27	1.544 (2)	C22—H22A	0.9700
C8—C9	1.569 (2)	C22—H22B	0.9700
C8—C14	1.577 (2)	C24—H24A	0.9600
C9—C11	1.538 (2)	C24—H24B	0.9600
C9—C10	1.573 (2)	C24—H24C	0.9600
C9—H9A	0.9800	C25—H25A	0.9300
C10—C19	1.540 (2)	C25—H25B	0.9300
C11—C12	1.538 (2)	C26—H26A	0.9600
C11—H11A	0.9700	C26—H26B	0.9600
C11—H11B	0.9700	C26—H26C	0.9600
C12—C13	1.520 (2)	C27—H27A	0.9600
C12—H12A	0.9700	C27—H27B	0.9600
C12—H12B	0.9700	C27—H27C	0.9600
			
C3—O2—H1O2	109.5	C13—C14—C18	110.67 (13)
C23—O3—C20	110.86 (14)	C15—C14—C8	117.57 (12)
C2—C1—C10	118.06 (15)	C13—C14—C8	109.68 (14)
C2—C1—H1A	107.8	C18—C14—C8	111.65 (12)
C10—C1—H1A	107.8	C14—C15—C16	102.95 (13)
C2—C1—H1B	107.8	C14—C15—H15A	111.2
C10—C1—H1B	107.8	C16—C15—H15A	111.2
H1A—C1—H1B	107.1	C14—C15—H15B	111.2
C3—C2—C1	114.24 (15)	C16—C15—H15B	111.2
C3—C2—H2A	108.7	H15A—C15—H15B	109.1
C1—C2—H2A	108.7	C15—C16—C17	105.76 (14)
C3—C2—H2B	108.7	C15—C16—H16A	110.6
C1—C2—H2B	108.7	C17—C16—H16A	110.6
H2A—C2—H2B	107.6	C15—C16—H16B	110.6
O1—C3—O2	121.97 (16)	C17—C16—H16B	110.6
O1—C3—C2	123.28 (17)	H16A—C16—H16B	108.7
O2—C3—C2	114.73 (14)	C20—C17—C13	116.35 (15)
C25—C4—C26	120.40 (15)	C20—C17—C16	111.16 (14)
C25—C4—C5	118.84 (15)	C13—C17—C16	104.56 (13)
C26—C4—C5	120.56 (15)	C20—C17—H17A	108.2
C4—C5—C6	110.90 (13)	C13—C17—H17A	108.2
C4—C5—C10	115.86 (15)	C16—C17—H17A	108.2
C6—C5—C10	111.62 (12)	C14—C18—H18A	109.5
C4—C5—H5A	105.9	C14—C18—H18B	109.5
C6—C5—H5A	105.9	H18A—C18—H18B	109.5
C10—C5—H5A	105.9	C14—C18—H18C	109.5
C5—C6—C7	111.55 (13)	H18A—C18—H18C	109.5
C5—C6—H6A	109.3	H18B—C18—H18C	109.5
C7—C6—H6A	109.3	C10—C19—H19A	109.5
C5—C6—H6B	109.3	C10—C19—H19B	109.5
C7—C6—H6B	109.3	H19A—C19—H19B	109.5
H6A—C6—H6B	108.0	C10—C19—H19C	109.5
C6—C7—C8	113.22 (15)	H19A—C19—H19C	109.5
C6—C7—H7A	108.9	H19B—C19—H19C	109.5
C8—C7—H7A	108.9	O3—C20—C24	105.58 (15)
C6—C7—H7B	108.9	O3—C20—C17	108.60 (13)
C8—C7—H7B	108.9	C24—C20—C17	114.09 (14)
H7A—C7—H7B	107.7	O3—C20—C21	103.40 (14)
C7—C8—C27	108.07 (13)	C24—C20—C21	112.04 (13)
C7—C8—C9	108.67 (12)	C17—C20—C21	112.27 (15)
C27—C8—C9	112.73 (14)	C22—C21—C20	102.91 (16)
C7—C8—C14	110.24 (14)	C22—C21—H21A	111.2
C27—C8—C14	109.77 (12)	C20—C21—H21A	111.2
C9—C8—C14	107.36 (12)	C22—C21—H21B	111.2
C11—C9—C8	112.00 (12)	C20—C21—H21B	111.2
C11—C9—C10	113.94 (14)	H21A—C21—H21B	109.1
C8—C9—C10	115.97 (12)	C23—C22—C21	104.06 (14)
C11—C9—H9A	104.5	C23—C22—H22A	110.9
C8—C9—H9A	104.5	C21—C22—H22A	110.9
C10—C9—H9A	104.5	C23—C22—H22B	110.9
C19—C10—C1	104.15 (14)	C21—C22—H22B	110.9
C19—C10—C5	112.46 (13)	H22A—C22—H22B	109.0
C1—C10—C5	109.68 (12)	O4—C23—O3	122.54 (18)
C19—C10—C9	113.76 (12)	O4—C23—C22	127.84 (17)
C1—C10—C9	109.96 (12)	O3—C23—C22	109.62 (17)
C5—C10—C9	106.81 (13)	C20—C24—H24A	109.5
C9—C11—C12	112.96 (15)	C20—C24—H24B	109.5
C9—C11—H11A	109.0	H24A—C24—H24B	109.5
C12—C11—H11A	109.0	C20—C24—H24C	109.5
C9—C11—H11B	109.0	H24A—C24—H24C	109.5
C12—C11—H11B	109.0	H24B—C24—H24C	109.5
H11A—C11—H11B	107.8	C4—C25—H25A	120.0
C13—C12—C11	108.90 (13)	C4—C25—H25B	120.0
C13—C12—H12A	109.9	H25A—C25—H25B	120.0
C11—C12—H12A	109.9	C4—C26—H26A	109.5
C13—C12—H12B	109.9	C4—C26—H26B	109.5
C11—C12—H12B	109.9	H26A—C26—H26B	109.5
H12A—C12—H12B	108.3	C4—C26—H26C	109.5
C12—C13—C14	111.86 (12)	H26A—C26—H26C	109.5
C12—C13—C17	119.33 (13)	H26B—C26—H26C	109.5
C14—C13—C17	104.96 (14)	C8—C27—H27A	109.5
C12—C13—H13A	106.7	C8—C27—H27B	109.5
C14—C13—H13A	106.7	H27A—C27—H27B	109.5
C17—C13—H13A	106.7	C8—C27—H27C	109.5
C15—C14—C13	100.31 (12)	H27A—C27—H27C	109.5
C15—C14—C18	106.36 (14)	H27B—C27—H27C	109.5
			
C10—C1—C2—C3	179.42 (14)	C12—C13—C14—C18	61.31 (17)
C1—C2—C3—O1	−167.70 (14)	C17—C13—C14—C18	−69.49 (15)
C1—C2—C3—O2	14.2 (2)	C12—C13—C14—C8	−62.31 (16)
C25—C4—C5—C6	−127.01 (19)	C17—C13—C14—C8	166.89 (12)
C26—C4—C5—C6	47.9 (2)	C7—C8—C14—C15	−69.81 (18)
C25—C4—C5—C10	104.4 (2)	C27—C8—C14—C15	49.1 (2)
C26—C4—C5—C10	−80.6 (2)	C9—C8—C14—C15	171.98 (15)
C4—C5—C6—C7	169.65 (14)	C7—C8—C14—C13	176.54 (12)
C10—C5—C6—C7	−59.54 (18)	C27—C8—C14—C13	−64.53 (17)
C5—C6—C7—C8	57.01 (18)	C9—C8—C14—C13	58.33 (15)
C6—C7—C8—C27	71.31 (17)	C7—C8—C14—C18	53.50 (18)
C6—C7—C8—C9	−51.32 (17)	C27—C8—C14—C18	172.43 (15)
C6—C7—C8—C14	−168.72 (12)	C9—C8—C14—C18	−64.71 (17)
C7—C8—C9—C11	−174.60 (12)	C13—C14—C15—C16	−45.09 (16)
C27—C8—C9—C11	65.63 (16)	C18—C14—C15—C16	70.21 (16)
C14—C8—C9—C11	−55.38 (17)	C8—C14—C15—C16	−163.85 (15)
C7—C8—C9—C10	52.28 (18)	C14—C15—C16—C17	31.41 (17)
C27—C8—C9—C10	−67.49 (18)	C12—C13—C17—C20	87.32 (18)
C14—C8—C9—C10	171.50 (13)	C14—C13—C17—C20	−146.36 (14)
C2—C1—C10—C19	173.23 (14)	C12—C13—C17—C16	−149.67 (15)
C2—C1—C10—C5	−66.19 (17)	C14—C13—C17—C16	−23.35 (15)
C2—C1—C10—C9	50.98 (19)	C15—C16—C17—C20	121.33 (15)
C4—C5—C10—C19	58.99 (18)	C15—C16—C17—C13	−4.99 (17)
C6—C5—C10—C19	−69.22 (17)	C23—O3—C20—C24	94.59 (15)
C4—C5—C10—C1	−56.40 (17)	C23—O3—C20—C17	−142.66 (14)
C6—C5—C10—C1	175.39 (12)	C23—O3—C20—C21	−23.24 (16)
C4—C5—C10—C9	−175.53 (12)	C13—C17—C20—O3	−61.86 (17)
C6—C5—C10—C9	56.25 (15)	C16—C17—C20—O3	178.64 (13)
C11—C9—C10—C19	−62.02 (18)	C13—C17—C20—C24	55.6 (2)
C8—C9—C10—C19	70.2 (2)	C16—C17—C20—C24	−63.92 (19)
C11—C9—C10—C1	54.34 (17)	C13—C17—C20—C21	−175.56 (15)
C8—C9—C10—C1	−173.43 (13)	C16—C17—C20—C21	64.93 (18)
C11—C9—C10—C5	173.30 (12)	O3—C20—C21—C22	29.77 (16)
C8—C9—C10—C5	−54.48 (16)	C24—C20—C21—C22	−83.45 (19)
C8—C9—C11—C12	55.20 (17)	C17—C20—C21—C22	146.64 (15)
C10—C9—C11—C12	−170.68 (12)	C20—C21—C22—C23	−26.18 (17)
C9—C11—C12—C13	−54.70 (17)	C20—O3—C23—O4	−174.19 (16)
C11—C12—C13—C14	58.51 (17)	C20—O3—C23—C22	6.50 (18)
C11—C12—C13—C17	−178.51 (14)	C21—C22—C23—O4	−166.08 (19)
C12—C13—C14—C15	173.32 (12)	C21—C22—C23—O3	13.18 (18)
C17—C13—C14—C15	42.51 (14)		

### Hydrogen-bond geometry (Å, °)

**Table:**

*D*—H···*A*	*D*—H	H···*A*	*D*···*A*	*D*—H···*A*
O2—H1O2···O1^i^	0.82	1.88	2.6541 (18)	156

Symmetry codes: (i) −*x*+2, *y*−1/2, −*z*+2.
